# Small and light arms violence reduction as a public health measure: the case of Libya

**DOI:** 10.1186/s13031-018-0162-0

**Published:** 2018-07-09

**Authors:** Gemma Bowsher, Patrick Bogue, Preeti Patel, Peter Boyle, Richard Sullivan

**Affiliations:** 1 Conflict and Health Research Group, King’s College London, London, UK; 2 Department of War Studies King’s College London, London, UK; 3 International Prevention Research Institute, Lyon, France; 4 King’s Centre for Global Health and Health Partnerships, Suite 2.13 Weston Education Centre, Cutcombe Road, London, SE5 9RJ UK

**Keywords:** Small and light weapons, Armed Libya

## Abstract

The conflict environment in Libya is characterized by continued pervasive insecurity amidst the widespread availability of small arms and light weapons (SALW). After the First Civil War, armed brigades took the law into their own hands and the resulting violence terminated a short-lived post-conflict period that has relapsed into a Second Civil War. The Libyan government has struggled to assert authority over armed groups and these brigades, refusing to disarm have contributed directly the initiation of a second conflict; some are motivated by self-defense, status, criminality, vindication or political aims.

Once, a bastion of public health in the Middle East and North Africa (MENA), the country now faces a substantial and unprecedented challenge: to rebuild a devastated health system amidst the burden of armed violence and the proliferation of small and light weapons (SALW) especially firearms of various kinds. The health system in Libya is compromised; healthcare professionals have little time to record or document such cases given the immediate clinical needs of the patient. This corresponding decreased capacity to deal with an increasing demand on services caused by SALW-related morbidity compounds the challenge of data collection and indicates that external support and advocacy are required.

A public health strategy towards effective SALW armed violence reduction and injury prevention requires the interdisciplinary advocacy of practitioners across the fields of justice, security, development, health and education. Through surveillance of firearms and injuries in the post-conflict environment we can better evaluate and respond to the burden of armed violence in Libya. In order to reduce armed a reconceptualisation of arms reduction campaigns must occur. Notable emerging evidence recommends the inclusion of community-based interventions and development programs which address local motivations for firearms ownership alongside improved international coordination. This renewed approach holds importance for recovery, development and securing the transition to peace.

The high prevalence of firearm ownership, weak institutions, nascent security forces, porous borders, inadequate weapons stockpiles, combined with high military spending, compounds public weaponisation as a health crisis for the entire MENA region.

## Background

The conflict environment in Libya is characterized by a widespread availability of small arms and light weapons (SALW). Once a bastion of public health in the Middle East and North Africa, the country now faces a substantial challenge: to rebuild a devastated health system amidst the scourge of SALW proliferation and relapsing civil war [57]. The International Crisis group has reported that over 125,000 weapons were in the hands of civilians by the end of the civil war ([22] p. i).

Libya is a middle-income country, with a pre-war population of approximately 6.4 million people [67], of which around 80% of its citizens reside within urban proximities [68]. Prior to the armed conflict of 2011 there were high rates of unemployment and appreciable socioeconomic inequality ([59, 68] pp.92–93). Despite decades of UN sanctions and international isolation hampering economic growth, the pre-war life expectancy at birth was 75 and comprehensive access to health services was achieved [67]. Quite rightly, Libya’s health system was hailed as a public health success story [57].

No single policy can address health in a complex and relapsing conflict environment such as Libya [41]. Violence as a complex phenomenon is intrinsically linked to existing social circumstances [6, 9]. Buvinic and Morrison’s context [8] analysis of the determinants of violence cites income inequality, poverty, availability of weapons and weak institutional capacity as notable common factors when applied in the Libyan context. Recognition of a coordinated interdisciplinary policy strategy is therefore necessary in addressing and managing SALW. Armed violence prevention requires the advocacy of interdisciplinary practitioners across the fields of criminal justice, public health, anthropology, journalism, human rights and education ([4] p.28, [26, 34]).

## A history of armed conflict in Libya

Since 2011, the health system of Libya has been heavily affected by armed conflict. The Libyan uprising and the First Civil War was an eight-month internal armed conflict lasting from 17th February to 23rd October, 2011. It officially ended with the killing of Muammar al-Gaddafi, the former leader of Libyan Arab *Jamahiriya* [24]. The civil conflict was characterised by widespread violence against civilians, characteristic of what Duffield [14] has termed ‘new wars’. The most fervently loyal military units were fighting alongside international mercenaries. These units were given license to fire upon unarmed civilian populations protesting in the eastern city of Benghazi, igniting revolutionary grievances and ultimately limiting any international political solution following UN Security Council Resolution 1973.

The armed insurrection was subsequently supported by a NATO ‘Right to Protect’ (R2P) operation under the auspices of Resolution 1973. This international military intervention commenced in order to protect civilians for humanitarian reasons following Gaddafi’s televised *‘zanga zanga’* speech which incited ‘street by street’ cleansing [68]. The aim of the R2P NATO operation was to control Libyan airspace and prevent fighter-jets attacking civilians, as occurred in Benghazi. Consequently, the withdrawal of Libyan forces from the eastern cities of Tobruk and Benghazi left rebel militias with open access to abandoned extensive SALW caches, such as those in the city of Ajdabiya. From February 2011 onwards, the flows of SALW increased through numerous trading and trafficking routes, with most arms funded and acquired initially by local anti-regime patrons [36].

By the end of the first period of the conflict there was in excess of 125,000 SALW in the hands of militias ([22] p.i). After the fall of Gaddafi, several thousand well-armed Tuareg fighters who fought as mercenaries further contributed directly to the 2012 conflict in the Azawad region of Northern Mali [68]. A 2013 UN Security Council report [60] indicates that other non-state actors were armed with weapons from Libya in at least twelve countries across the Middle East and North Africa (MENA). A second period of conflict erupted in 2014 after a prolonged period of insurgency, fuelled by SALW proliferation, between the democratically elected Council of Deputies, known here as the Libyan government, and the Islamist government of the General National Congress. The UN brokered a ceasefire in 2016, which has not been ratified by all parties. Whilst a new government attempts to assert itself in the face of rival factions, the continued widespread violence involving SALW threatens ongoing prospects for a stable peace.

## Consequences of SALW on Libyan public health

The impacts of violent armed conflict in Libya are long-term; victims are usually young males but also include other vulnerable groups, particularly those of Tawerghan or sub-Saharan African background [2]. The pervasive impact of SALW availability is not just confined to combat-related deaths but it also adversely impacts the prospective post-conflict period through injuries caused by violence, negligent discharge and aerial firing of SALW [38].

SALW proliferation is a dangerous consequence of armed conflict. It is further facilitated through illicit trafficking in combination with inadequate disarmament, demobilization and reintegration (DDR) programs. While the proliferation of arms is not considered the cause of violence, it does increase the severity of violence as a force multiplier ([47] p.113). The direct consequences of armed violence pose multifaceted challenges for the health system [62]. The economic and human costs significantly burden resource-limited services [29]. This correlates with a reduced capacity for quality health service provision in the post-conflict environment [64]. To illustrate the magnitude of health-costs, one government policy for treating ‘war-disabled’, estimates that for services provided by Jordan, expenditure ranged from USD 400 million per year [50]. We currently possess no similar data on the extent of healthcare professional loss in Libya.

The indirect effects of armed violence include the disruption of livelihoods through population displacement leading to pervasive insecurity [26, 62]. Moreover, other factors such as psychological trauma, inadequacy of essential services amid resurgence in vaccine-preventable disease adversely affect security, health and development [30, 43, 62]. These indirect effects may also add to growing population discontentment with the newly appointed government, causing the significant continued insecurity.

A range of pragmatic local interventions with the aim of mitigating the destructive consequences of violence can then be achieved [39]. In Libya, this also holds relevance via ‘peace through health’ dividends, as further relapse into armed conflict may be prevented [4]. The importance of adequate disarmament, demobilisation and reintegration (DDR) and weapons reduction programs in reducing the supply of SALW has been emphasized as ‘best practice’ by the UN and World Bank [38]. Although there remains limited evidence of effectiveness [39], addressing SALW as a violent ‘force multiplier’ is integral for the achievement of a reduction in the ‘heterogeneous forms of violence’ [40]. SALW have clearly played a role in exacerbating and facilitating human rights atrocities, including torture, sectarian and interpersonal [47]. Other supply-focused interventions include UN embargoes, which have had variable success, are notably absent in the Libyan case.

## Armed violence reduction as a public health issue

SALW control is increasingly recognised as a public health good, especially in the post-conflict environment ([17, 31] pp. 228–34). The expected benefits in violence reduction far outweigh the costs across a number of indicators ([31] p.18). Achieving reduction in armed violence involves a variety of prevention strategies, in addition to SALW control, that aim to reduce levels of mortality and morbidity [17, 30, 52]. Political will and civil society advocacy for SALW reduction have been evident with large collaborative campaigns such as ControlArms which is aimed towards establishing policy which addresses the root causes of armed violence [12, 62].

In Libya, in contrast to neighbouring Egypt or Tunisia, governance and institutional structures to implement SALW control policies were either seriously weakened or virtually non-existent following the First Civil War. The sociopolitical context in Libya has been ‘local, atomised’ and tribal’ [16]. For Libya, the local and tribal, ethnic, political and religious systems may provide a unique opportunity for implementation of community-level interventions [33].

### SALW: Measuring the public health impact

Measuring the public health impact of SALW poses difficulties in the post-conflict environment when surveillance data are sparse [58], particularly for violence against specific groups such as women ([30] p.138). Public health reporting systems are still lacking, without any needs assessment published to date [57]. The surveillance system identified for review here is one that involves the health system reporting of firearms injuries. The importance of public health reporting systems i.e. to collect injury surveillance data with the use of existing tools for injury prevention action is well-established [65]. By improving the recognition of recording SALW mortality and morbidity a crucial step is made towards understanding the root causes and subsequently targeting of interventions [37]. Other outcome variables to consider are crime rates, attitudes and beliefs. Economic data such as the direct medical costs of providing health services may also provide measurable outcomes toward the assessment of burden.

To illustrate the additional burden of disease accrued through armed violence, gunshot injuries may either result in mortality or lead to disability and psychological distress. The most severe injuries are exemplified by spinal and traumatic brain injuries resulting in loss of function and accounting for an especially significant burden on medical services, lost productivity and opportunity cost (Sadeq Institute, 2012) [21, 48]. Rehabilitation services are often inadequate and prohibitively expensive ([31] pp. 35–6).

Before the onset of the 2011 civil war, Libya’s homicide rates were at 2.9 per 100,000 people. Accurate homicide figures since the onset of the war are not known. Although the International Institute for Strategic Studies (IISS) reports 30,000 combat-related deaths in 2011 and 828 deaths in 2012 [24], which would indicate that Libya’s violence-related mortality rate is greater than 13 per 100,000, of which a significant number will have been through SALW.

### Socio-demography and demand: drivers of SALW proliferation

Recorded data quantifying the extent of availability of SALW in Libya is uncoordinated and varying in quality. Initiatives to convert these recordings into a centralised dataset would allow for increased oversight [18]. Prior to the civil war, the numbers of firearms held by civilians were estimated to be over 906,000, corresponding to 15.5 firearms per 100 people [27].

**Table 1:** Reccommendations for SALW Violence Reduction in Libya

Reccommendation 1. Establish a public health reporting system for SALW associated injuries 2. Denormalise the arms industry and emphasise the norms upheld by the ATT 3. Support regional cooperation to effect reductions in military spending 4. Encourage ECOWAS to uphold an enforced moratorium on import, export and manufacture of small arms in the region 5. Develop local and community interventions engaged with mitigating individual motivations for SALW possession 6. Reinsert armed brigade revolutionaries into GNC forces 7. Enlist existing community leaders such as brigade commanders in SALW reduction programmes 8. Initiate demand-side DDR initiative such as community-based financing, loan exchanges and weapons lotteries 9. Establish legislation targeting SALW reduction through channels such as firearm registration, age limits, waiting periods and background checks 10. Limit the scope illicit trafficking from neighbouring countries by enacting customs controls and vigilant policing	

The price of SALW is correlated with increased availability, and evidence suggests that cheap and plentiful guns increase the risk of violence ([20] Ch. 7). In 2011, a Kalashnikov could be bought for USD 250 in Libya [66]. The cost of a rifle is a proxy indicator for availability of SALW [28]. The average military spend of a neighbouring country is also used as a good indicator of cheap AK-47 availability [28]. In some areas of the Sahel, the AK-47 is accessible to anyone in the position to barter in return for essential commodities. So accounting for confounding factors, the risk for Libya is that cheap weapons availability is independently associated with an increased risk of relapse to armed conflict [54].

In Libya there are approximately 147 separate rival tribes and hundreds of different armed groups. Consequently, this creates a very complex security environment. The potential is clear; Duquet [15] recognises strong leadership as an important tipping factor in the analysis of armed violence in the Niger delta. The current security picture sees the Libyan Government seeking to assert control over a disparate selection of militias from which a coherent Libyan National Army may materialise. The commanders of brigades were shaped into efficient and experienced leaders under pressure and it may therefore be more pragmatic to conceptualise them as an asset rather than a threat [36]. In Libya, it will be a priority to either facilitate the disarmament and reintegration of armed-groups into whatever emerges as a unified national army – a prospect yet to emerge given the ongoing political ruptures. A G8 support package supplied by Italy, France and the UK defence forces to improve security has provided funding towards this.

Libya’s relatively homogenous population is at risk of fragmentation along ethnic, tribal and federal fault lines. Escalating tensions and mistrust between black Africans and Berbers in Kufra for example have resulted from their respective stereotype as either pro- or anti-Gaddafi [2]. Newly constructed and imposed collective identities incite fear [55]. As a consequence the motivations for weapons acquisition appear to be stable or increasing [38].

The high rates of ex-combatant unemployment combine with the prestige status afforded to SALW owners. This problem relates to the cultural and symbolic status the weapon has within society [46]. Under Gaddafi’s regime, the constitutional slogan ‘Power, wealth and weapons, in the hands of the people’ exemplified the idea of a ‘people’s militia’. A UNDP survey of perceptions of public safety indicated that 91% of Libyans felt safe in these conditions ([59] p.175). The present inability of the government to provide assurance of security and prevent armed groups from ‘operating above the law’ has further encouraged individual citizens to secure weapons for themselves in order to protect themselves ([23] p.35).

Corresponding poverty through lack of employment and failure of reintegration may also lead ex-combatants to armed violence as a means of livelihood [13]. Trafficking and criminality flourish as an additional side effect of conflict [8], without investment in employment or education, prospects for young men especially not be cultivated and the ‘combined effect of deprivation and increased weaponisation of the public’ risk driving a return to conflict [57].

## Public health interventions for SALW violence reduction

The relapsing conflict environment in Libya is characterized by the widespread availability of SALW. The new Government of National Accord now faces the substantial challenge of rebuilding a health system amidst ongoing political tensions and the burden of significant SALW-related injuries and disability. Recognition of a coordinated interdisciplinary policy strategy is therefore necessary in addressing and managing this preventable issue [17]. Table 1 presents ten key recommendations preceding a discussion of prospective public health initiatives to reduce SALW violence during and beyond the Libyan conflict. These recommendations constitute a range of successful interventions made elsewhere, and a few obvious areas for reform in the Libyan context specifically (Table 1).

### International arms control

The 2006 *Geneva declaration on Armed Violence and Development* was aimed at supporting state actors and civil society organisations to reduce the burden of armed violence [30]. This built on strong representation from the international community through the annual state reports by the UN Programme of Action to Prevent, Combat and Eradicate the illicit trade SALW. The comparative success of landmine reduction through the 1997 ‘Mine ban treaty’ provided the basis for this movement [42, 49].

Prior to 2013, the arms trade was regulated by the Programme of Action, and regional controls. The 2013 Arms Trade Treaty (ATT) is the first international legally-binding instrument to control transfer of conventional arms. The treaty is intended to limit conflict diversion, corruption and improve responsibility for arms deals. An ATT which controls the availability and transfer of small arms is important, but necessitates international enforcement procedures to ensure the legitimacy of the ATT.

Successful comparisons for injury prevention must be grounded in previous public health experience. Pinto et al. [47] argue for the denormalisation of the arms industry in similar respects to what was seen with the progressive control and restrictions placed on the tobacco industry [37, 47]. The countering of the powerful lobby behind both tobacco and automobile industries demonstrates the efficacy of public health advocacy for regulatory-change ([4] p. 3–12). Multinational government and commercial interests may be enticed to make concessions since SALW constitutes only a small part of the lucrative export market. The major profits for the international community are likely to be demonstrated by a reduction in the burden of armed violence [61].

Multilateral trade commitments for conventional arms that pledge minimum standards for responsible trade are a promising avenue for the reduction in the supply of SALW to non-state actors. The ATT contains a categorical prohibition on the transfer of SALW and ammunition to a state which may be capable of commission of genocide, crimes against humanity or war crimes [35]. The treaty has its limitations, since it does not limit the production of SALW, thus incentive-to-use still maintains existing market demand. Similarly, it ignores the fact that arms are increasingly being produced by craft industries in Africa [56]. Further, it does nothing to prohibit transfers to non-state actors, nor does not clarify how states will prevent unintentional diversion of arms to illicit traders, such as the infamous 2008 shipment aboard the MV *Faina* which was hijacked by Somali pirates [56].

### Regional cooperation for armed violence reduction

Collier [10] states that ‘arms embargoes can be made to work’ and cites the 1998 moratorium on import, export and manufacture of small arms between the Economic Community of West African States (ECOWAS) as an example. In this case, failure to reduce armed violence was probably due to its limited scope as a voluntary moratorium prior to 2006. It follows that with adequate regional collaboration of North African states an enforced moratorium has scope to improve outcomes in this sphere. After all, the ECOWAS moratorium did not address the potential for illicit supplies from the porous MENA countries following the Arab Spring, nor the burgeoning African craft industry in states such as Ghana which has the potential to produce 200,000 SALW per year ([56] p.20). The consideration of the distribution system of traders and middle-men in this market is important for the implementation of any regulatory monitoring process [47].

A highly fragile security environment has emerged in the Sahel [56]. The mobilisation of support amongst all regional actors is important for armed violence reduction. Renewed national, regional and multi-lateral co-ordination for the reduction in military spending holds significant promise. Collier optimistically identified the role of a neutral enforcer i.e. the UN to enforce such a pan-African enforced reduction in military spending so as to protect fragile states from conflict. It is up to the international community to address the Libyan context as an opportunity for action in the Sahara-Sahel region.

### Military spending in the post-conflict environment

Collier’s economic analysis through statistical modeling indicates that military spending is correlated with increased risk of re-entry into conflict [10]. Cost-effective interventions for prevention of re-entry include peacekeeping and an ‘over-the-horizon’ international technical support such as that deployed by the UK in Sierra-Leone ([11] Ch.4). Currently Libya lacks either of these initiatives. Targeting regional commitments towards reductions in military spending for reducing risks could be effective an effective public health strategy for the reduction of consequent violence.

Military spending in fragile-states is excessive by any standards. Collier’s [10, 45] statistical analyses demonstrate that military spending is counter-productive, precipitating the violence it is meant to deter. Only by including a SALW-reduction programme can a sustainable reduction in armed violence be achieved an SALW control may further be seen as a precondition towards broader health and equity promotion strategies.

### Leadership

**Fig. 1:**
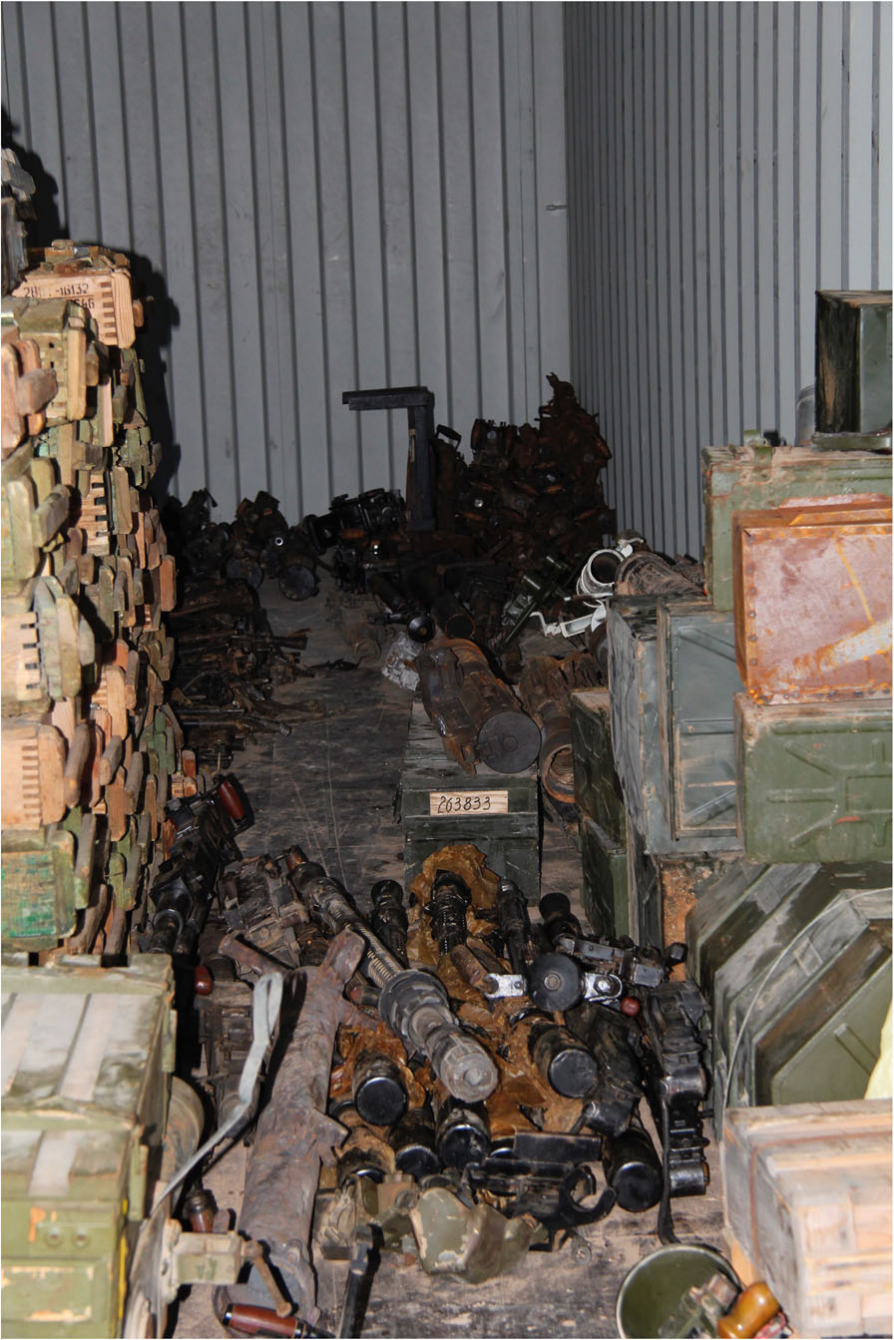
Brigade weapons Stockpile. Picture Credit Richard Sullivan 2011

**Fig. 2:**
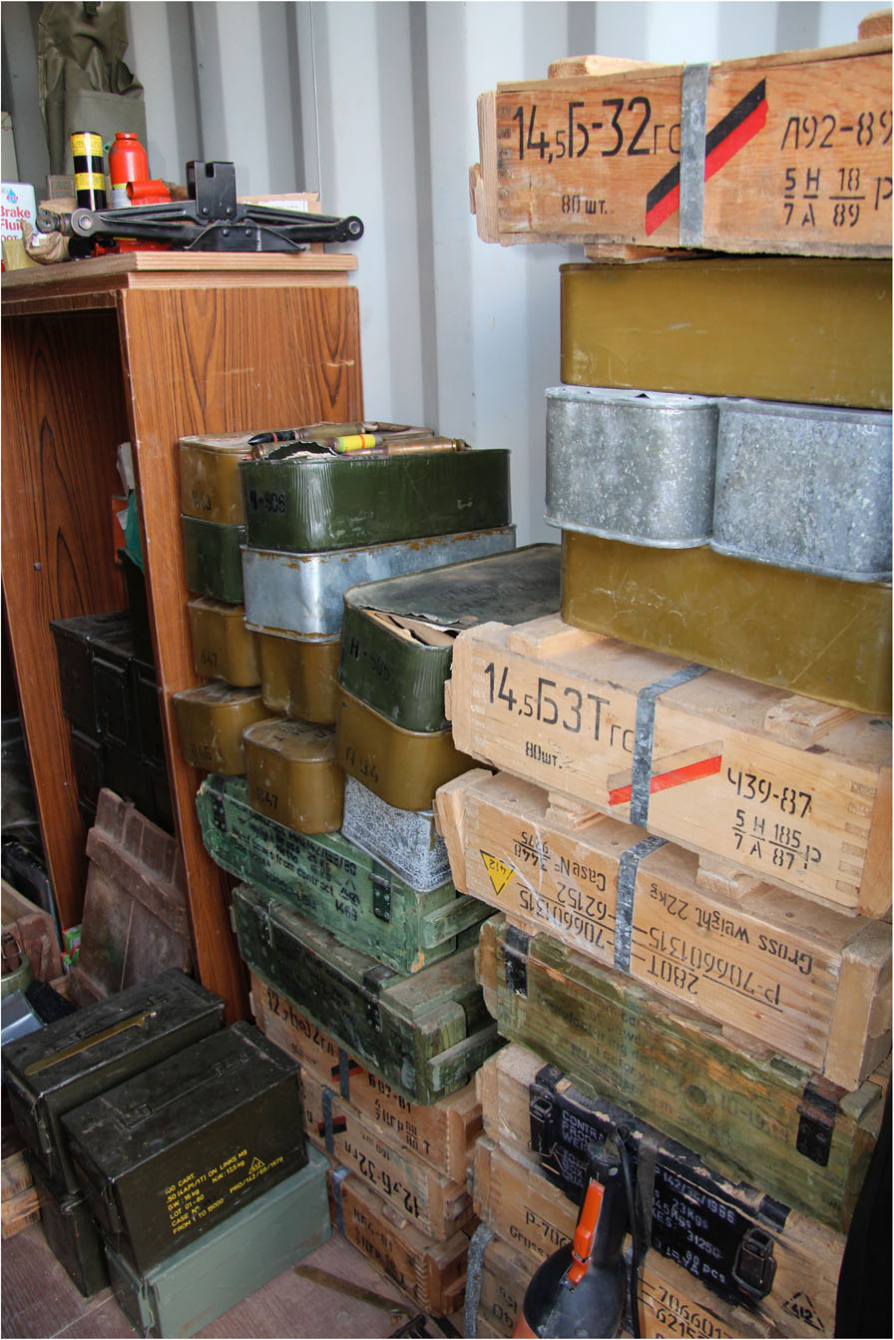
Brigade Weapons Stockpile. Picture Credit Richard Sullivan 2011

**Fig. 3:**
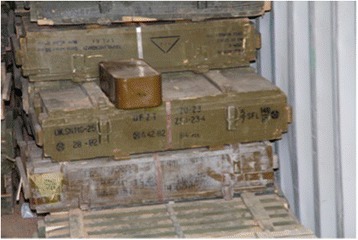
Brigade Weapons Stockpile. Picture Credit Richard Sullivan 2011

There is potential for brigade commanders to exercise their authority for the disarmament of SALW and reintegration of their combatants – provided of course that some compromises be made [36]. Political difference between brigades and their governing factions must be firmly settled as part of the process of statebuilding. Nevertheless, the hierarchies established within these groups have already assisted in achieving weapons registration systems with a varying degree of sophistication, as well as some centralised control of weapons stockpiles during the period between civil wars [16]. Accountability within the hierarchical structures of the revolutionary brigades is a potential asset that could be harnessed for encouraging declaration of SALW ownership preceding a national registration or licensing system. Figures 1, 2 and 3 are photographs of a weapons stockpile of a brigade.

Community organizational hierarchies pose a vital opportunity to motivate former commanders and elders to implement measures to reduce SALW violence. Such initiatives have already been proven effective in Colombia; an effective alternate-day public ban on carrying firearms was enforced in Bogota and was associated with a reduction in homicide rates [63]. Correspondingly, the Institute of Migration (IOM) supported representative community-policing through the Kosovo Protection Corps, which demonstrated successes in violence reduction that may be transferable to the Libyan situation [25].

### Disarmament, demobilisation and reintegration (DDR)

Weapons reduction programs combined with DDR show successes when they are adequately prioritized [38]. The International Crisis Group ([23], p.37–8) reports that during the period of insurgency between the first and second civil war, armed groups were emboldened by the slow progress of the GNC, vindicating their perceived importance in filling the ‘security vacuum’ and undermining the authority of the state. A robust political solution must be brokered between the two major rival governments before a feasible national DDR programme may be successfully implemented in order to avoid such vacuums that encourage the proliferation and usage of SALW. Such a programme must be a priority for post-conflict health system reconstruction efforts in order to prevent the negative consequences of persistent insecurity posed by potential militias.

### Targeted demand –side interventions

SALW supply reduction through DDR campaigns forms an integral component of conflict management strategies [38]. Demand-side reduction interventions focusing on the firearm ownership include a diverse range of development and institutional strengthening measures as well as measures aimed at reducing the ‘culture of violence’ [39]. Arya and Cukier [3] argue that an effective intervention will ‘strike at the roots of the disease’ – the risk factors identified are as follows: high prevalence of firearm ownership, high military spending in the region, brigade impunity and lack of stockpile and border security.

Voluntary cash incentives such as those used in buy-back programs in post-conflict Sierra Leone and Liberia should be approached with caution. Despite the successful destruction of thousands of SALW [56], some argue that such buy-back programs focus solely on individuals and align primarily with development agencies’ narrow metrics success measured as numbers of weapons collected [38]. Despite labels of ‘quick wins’ and ‘best practice’ espoused by WHO and the World Bank; the extent to how conventional DDR programs reduce SALW availability is ‘largely unknown’ [39].

Expanded efforts with community-based finance initiatives have shown major successes in Central America and the Caribbean ([31, 39] p.27). ‘Weapons for development’ programs focus on the community rather than the individual [38]. Moreover, Alusala and Institute for Security Studies (South Africa) ([1] pp.41–3) argue that more innovative approaches which remove cash incentives from the equation are usually more appropriate. For instance, ‘weapons lotteries’ implemented in Mozambique and Bosnia involve returning SALW and winning an item, for example a DVD player or another similar commodity. This indicates that careful tailoring of dispute resolution programmes (DRPs) can be made in order to reduce SALW availability whilst avoiding corruption.

The reintegration of former Sierra Leonean RUF militias by exchanging firearms for loans has allowed former combatants to rent vehicles for use in employment as taxi drivers [44]. Former-combatants are thus reinserted into society through meaningful labour in exchange for giving up the tools of combat [5]. The use of cash loans can be seen to directly address some of the underlying factors that risk a fall back into conflict; namely unemployment and poverty. These kinds of finance initiatives work by enabling social mobility and cohesion through the abandonment of the means of violence. The idea is transferable to other occupations, instead of joining the LSF or SSC, the training of construction workers is another appropriate integration option, particularly relevant following the destruction of health infrastructure in Libya.

### Institutional capacity strengthening

Collective multisectoral reforms (UNDP, pp. 147–49) are vital for the initiation and maintenance of SALW reduction in Libya. This is challenging given that Libya’s institutions have undergone appreciable neglect over the 42-years of Gaddafi rule and 7 years of conflict and insurgency.

The establishment of integrated legislation focusing on armed violence reduction is required in the medium to long-term. Laws regarding registration of firearm ownership, restrictions such as age limits, waiting periods, and background checks have all proved effective to varying degrees ([20] Ch.8). The enactment and subsequent enforcement of such legislation will require political will from a strengthened National Government alongside the restoration of confidence through dialogue and support from civil society and tribal leaders. Improving and strengthening trust between citizens and newly emerging institutions is necessary. Despite their reputation as ‘Gaddafi-era relics’, the careful maintenance of the judicial system is a recognised priority ([23] pp.20–1).

### Border security

Cheap, durable, concealable weapons are flowing through Libya’s porous southern desert borders to where they are in highest demand. There were pre-existing illicit trafficking networks spanning from West Africa and spreading north towards the Mediterranean via the Tuaregs of the Sahel. Trafficking to and from the Maghreb; Algeria, Tunisia and Libya. The Libyan government has assured the UN that borders with Algeria, Chad, Niger, Sudan and Tunisia are now closed, and declared a restricted military zone in the southern region [60]. Reintegrated combatants are responsible for protecting the border crossings aiming to minimize the contagion effects of trafficking. The long-term cost of failure in securing borders involves a greater risk of criminal trafficking [18].

There is a notable difficulty in retrieving accurate data on arms transfers; especially those on the grey and illicit black market. This highlights the importance of collecting data on SALW flows, stockpiles and price. In Libya, years of UN and EU embargo deterred legal arms supply and may have initiated illicit trafficking and diversion from neighbouring countries [51]. Small Arms Survey [53] research highlights the importance of tracing marked weapons and serial numbers, allowing for identification of weapons suppliers and flows and addressing the source of cross-border proliferation of SALW [19, 51]. An important step towards border control would be to elaborate export and import controls through legislation and enforce these with customs inspections [7].

## Conclusion

Armed violence reduction and injury prevention requires the advocacy of interdisciplinary practitioners across the fields of criminal justice, security, development, health, policy, anthropology and education. Through surveillance of SALW and public health injury reporting we can evaluate the burden of armed violence in Libya. Kruk et al. [32] argue that this holds relevance in health and state-building dividends, as further relapse into armed conflict may be prevented.

Unfortunately, Libya demonstrates the severe health and human rights consequences that occur due to failure of these processes. We know that DDR and weapons reduction campaigns alone will not effectively reduce the demand for SALW. The post-conflict environment therefore requires a diverse package of DDR, second generation weapons reduction, SSR, judiciary and legislative interventions. At this time of great instability for the Libyan Government an SALW weapons reduction strategy holds importance for ‘recovery and development’ and securing the transition to peace [38]. In this way the public health and security sectors can effect a pragmatic partnership during and after the current period of insecurity that afflicts Libya.

## Abbreviations

ATT: Arms Trade Treaty
DDR: Disarmament, demobilization and reintegration
ECOWAS: Economic Community of West African States
EU: European Union
GNC: General National Congress
LSF: Libya Shield Forces
MENA: Middle East and North Africa
NATO: North Atlantic Treaty Organisation
PSSM: Physical security and stockpile management
R2P: Right to protect
RUF: Revolutionary United Front
SALW: Small and light weapons
SSC: Supreme security committee
UN: United Nations
WHO: World Health Organistion
